# Glucagon-Like Peptide-1 Mediates the Protective Effect of the Dipeptidyl Peptidase IV Inhibitor on Renal Fibrosis via Reducing the Phenotypic Conversion of Renal Microvascular Cells in Monocrotaline-Treated Rats

**DOI:** 10.1155/2018/1864107

**Published:** 2018-01-23

**Authors:** Jian Xu, Jingjing Wang, Yusheng Cheng, Xiang Li, Mengyu He, Jiali Zhu, Honghao Han, Guihong Wei, Hui Kong, Weiping Xie, Hong Wang, Xiangrong Zuo

**Affiliations:** ^1^Department of Respiratory & Critical Care Medicine, The First Affiliated Hospital of Nanjing Medical University, Nanjing, Jiangsu 210029, China; ^2^Department of Critical Care Medicine, The First Affiliated Hospital of Nanjing Medical University, Nanjing, Jiangsu 210029, China

## Abstract

Chronic kidney diseases are characterized by renal fibrosis with excessive matrix deposition, leading to a progressive loss of functional renal parenchyma and, eventually, renal failure. Renal microcirculation lesions, including the phenotypic conversion of vascular cells, contribute to renal fibrosis. Here, renal microcirculation lesions were established with monocrotaline (MCT, 60 mg/kg). Sitagliptin (40 mg/kg/d), a classical dipeptidyl peptidase-4 (DPP-4) inhibitor, attenuated the renal microcirculation lesions by inhibiting glomerular tuft hypertrophy, glomerular mesangial expansion, and microvascular thrombosis. These effects of sitagliptin were mediated by glucagon-like peptide-1 receptor (GLP-1R), since they were blocked by the GLP-1R antagonist exendin-3 (Ex-3, 40 ug/kg/d). The GLP-1R agonist liraglutide showed a similar renal protective effect in a dose-independent manner. In addition, sitagliptin, as well as liraglutide, alleviated the MCT-induced apoptosis of renal cells by increasing the expression of survival factor glucose-regulated protein 78 (GRP78), which was abolished by the GLP-1R antagonist Ex-3. Sitagliptin and liraglutide also effectively ameliorated the conversion of vascular smooth muscle cells (SMCs) from a synthetic phenotype to contractile phenotype. Moreover, sitagliptin and liraglutide inhibited endothelial-mesenchymal transition (EndMT) via downregulating transforming growth factor-*β*1 (TGF-*β*1). Collectively, these findings suggest that DPP-4 inhibition can reduce microcirculation lesion-induced renal fibrosis in a GLP-1-dependent manner.

## 1. Introduction

Renal fibrosis, resulting from infections, toxins, xenobiotics, mechanical obstruction, chronic infections, or autoimmune diseases [1], leads to a progressive, irreversible loss of functional renal parenchyma and, eventually, renal failure [2]. Almost all cell types in the kidney, including fibroblasts, pericytes, endothelial cells (ECs), vascular smooth muscle cells (SMCs), tubular epithelial cells, podocytes, and mesangial cells, as well as inflammatory cells such as lymphocytes and macrophages, are involved in the progress of renal fibrosis [3, 4]. Unquestionably, activated myofibroblasts are known to be the key effector cells in the pathogenesis of renal fibrosis as they are responsible for the exaggerated and uncontrolled production of fibrillar collagens and contractile proteins and ultimately result in excessive matrix deposition [5]. Most myofibroblasts or myofibroblast-like cells are derived from the transition of renal cell types [6]. Among them, 50% arise from local resident fibroblasts via proliferation, 35% are derived via differentiation from bone marrow, 10% result from endothelial-to-mesenchymal transition (EndMT), and 5% originate from epithelial-to-mesenchymal transition (EMT) [6]. Apart from those above, cells of the synthetic phenotype of SMCs, which are derived from the contractile SMC phenotype, can reflect both fibroblast and smooth muscle features, similar to myofibroblasts [7] and also participate in renal progressive vascular sclerosis and fibrosis [8, 9] by producing excessive matrix elements.

Microcirculation constituted by ECs and SMCs maintains the structure and function of the kidney via supplying nutrients and oxygen. The dysfunction, phenotypic conversion, and apoptosis of microcirculatory cells result in increased glomerular capillary wall permeability, the development of albuminuria and the sequential loss of normal renal function, including maintaining glomerular filtration, tubular reabsorption, and systemic recirculation of different substances [10]. Additionally, progressive loss of the normal renal microvasculature leads to hypoxia, which is a prominent determinant of chronic kidney diseases, ultimately promoting renal fibrogenesis. Renal fibrosis, resulting from microcirculation lesions, further worsens the physical function of the kidney [11]. Among microcirculation lesions, EndMT and failure of SMCs to maintain the contractile phenotype are the most important lesions as they can further promote profibrotic responses, scarring, chronic hypoxia, and the subsequent deterioration of renal function [12]. Therefore, protecting the kidney from fibrosis by alleviating microcirculation lesions such as EndMT and conversion of SMCs from the contractile to synthetic phenotype is emergent.

In recent years, multiple actions of dipeptidyl peptidase 4 (DPP-4) inhibitors and glucagon-like peptide-1 receptor (GLP-1R) agonists have been well defined in animal models of diabetic kidney disease (DKD) [13–15]. Meanwhile, several other experiments have explored the effects of DPP-4 inhibitors and GLP-1R agonists on non-DKD, including tacrolimus-induced kidney injury [16], adriamycin nephropathy [17], cisplatin-induced nephrotoxicity [18], renal ischaemia-reperfusion injury [19, 20], and ureteral obstruction-induced renal interstitial fibrosis [21]. In addition, GLP-1 is the most important substrate of DPP-4 and can mediate the multiple actions of DPP-4 inhibition in cardiovascular disease [22] and diabetes [23]. However, few studies have classified whether glucagon-like peptide-1 (GLP-1) mediates the protective effect of DPP-4 inhibitors on renal fibrosis, and most experiments focused on the effect of DPP-4 inhibitors and GLP-1 agonists have been on activated myofibroblasts derived from fibroblasts and EMT and infiltrated cells. Given the central role of microcirculation in the maintenance of renal structure and function, evaluating the potential of DPP-4 inhibition to reduce renal microcirculation lesions is important. Hence, the present experiment was performed to explore whether the DPP-4 inhibitor sitagliptin (SG) and GLP-1R agonist liraglutide (Li) have a protective effect against renal fibrosis caused by monocrotaline- (MCT-) induced renal microcirculation lesions [24] and whether the protective effect of DPP-4 inhibition on renal fibrosis is mediated by GLP-1.

## 2. Materials and Methods

### 2.1. Ethics Statement

All procedures in this study were carried out according to the National Institutes of Health Guide for the Care and Use of Laboratory Animals (publication number 85–23, revised 1996) and approved by the Animal Ethical and Welfare Committee of Nanjing Medical University (IACUC- 1601196).

### 2.2. Experimental Protocol

Eight-week-old male Sprague-Dawley rats (Shanghai B&K Laboratory Animal Company, Shanghai, China) that initially weighed 210–220 g were raised in cages under a controlled environment at the animal core facility of Nanjing Medical University. After acclimatization with free access to water and food for one week, rats were randomized to eight groups containing eight rats each. Rats in the control group (Con) were given an intraperitoneal (i.p.) injection of 0.2 ml saline, while the rats in the other groups were challenged with monocrotaline (MCT) (60 mg/kg, i.p., Sigma-Aldrich, St. Louis, MO, USA). On each of the following days, rats injected with MCT were treated with the DPP-4 inhibitor sitagliptin (SG, 40 mg/kg, gavage, Merck Sharp & Dohme, Australia) with (MCT + 40 mg/kg SG) or without the GLP-1R antagonist exendin 9-39 (exendin-3, Ex-3, 40 *μ*g/kg, i.p., Santa Cruz Biotechnology, MCT + 40 mg/kg SG + 40 *μ*g/kg Ex-3) or were given a subcutaneous injection of the GLP-1R agonist liraglutide (Li, Novo Nordisk A/S, Denmark) at a dose of 0.1 mg/kg (MCT + 0.1 mg/kg Li), 0.2 mg/kg (MCT + 0.2 mg/kg Li), 0.4 mg/kg (MCT + 0.4 mg/kg Li), or 0.8 mg/kg (MCT + 0.8 mg/kg Li). Meanwhile, rats in the Con group and MCT group received 0.9% saline (0.2 ml/200 g, gavage). The body weight of the rats was measured every other day to adjust the dose. Four weeks later, the rats were sacrificed, and kidneys were collected for western blot and histological analysis.

### 2.3. Morphological Evaluation

Collected kidneys were immediately fixed in 4% polyformaldehyde (pH 7.4) for 24 h, embedded in paraffin, and then sliced into 4–6 *μ*m sections. After haematoxylin and eosin (HE) staining, 10 glomeruli per rat were randomly chosen for calculating the glomerular surface area using Image-Pro Plus software 6.0. To evaluate the mesangial matrix area (%), periodic acid-Schiff (PAS) staining was employed according to the protocol [16]. Ten glomeruli from each rat were analysed on a digital microscope screen grid containing 540 points in Adobe Photoshop Element 6.0. The percentage of relative mesangial matrix area was calculated by (number of grid points in the mesangial area)/(total number of points in the glomerulus). To evaluate the degree of renal fibrosis, the Masson trichrome stain (MTS) was used as described previously [25]. The sample sections were photographed randomly with a Leica 2500 microscope (Leica Microsystems, Wetzlar, Germany), and the percent of fibrotic areas was quantified using Image-Pro Plus software 6.0.

### 2.4. Protein Extraction and Western Blot Analysis

Total proteins were extracted from the whole renal tissue with RIPA Lysis and Extraction buffer (Thermo Fisher Scientific, Middletown, VA, USA) containing a protease inhibitor cocktail (Roche Company, Germany) for western blotting. First, 30 mg protein was electrophoresed on an 8–12% SDS-PAGE minigel, and the proteins were transferred onto a polyvinylidene difluoride (PVDF) membrane. After that, the PVDF membranes were hybridized in 5% nonfat dry milk or 5% bovine serum albumin (BSA) for 1 h at room temperature and were incubated overnight at 4°C with antibodies against DPP-4 (1 : 1000, Abcam PLC, Cambridge, MA, USA), GLP-1 (1 : 1000, Abcam PLC), cleaved-caspase 3 (1 : 1000; Cell Signaling Technology, Danvers, MA, USA), glucose-regulated protein 78 (GRP78, 1 : 1000, ProteinTech Group, Inc., Chicago, IL, USA), Bax (1 : 1000; Cell Signaling Technology), Bcl2 (1 : 1000; Cell Signaling Technology), smooth muscle 22 alpha (SM22*α*, 1 : 1000, ProteinTech Group), von Willebrand factor (vWF, 1 : 1000, ProteinTech Group), *α*-smooth muscle actin (*α*SMA, 1 : 1000, Abcam PLC), transforming growth factor-*β*1 (TGF-*β*1, 1 : 1000; Cell Signaling Technology), TGF*β* receptor 1 (TGF*β*R1, 1 : 1000, Abcam PLC), Smad3 (1 : 1000; Cell Signaling Technology); phosphorylated Smad3 (1 : 1000; Cell Signaling Technology), Snail (1 : 1000; Cell Signaling Technology), bone morphogenetic protein receptor type 2 (BMPR2, 1 : 1000, Abcam PLC), and *β*-actin (1 : 5000, ProteinTech Group). The PVDF membrane was then incubated with a horseradish peroxidase-conjugated secondary antibody (1 : 10000, ProteinTech Group) for 60 min at room temperature. Specific signals were detected using a VersaDoc Imaging System (Bio-Rad, Hercules, CA). Band intensities were normalized to *β*-actin and then to the control for comparisons and were analysed densitometrically using NIH Image 1.46 software.

### 2.5. Immunohistochemistry

Immunohistochemistry was performed as described previously [25]. Immunohistochemistry for DPP-4 (1 : 100, Abcam PLC), caspase 3 (1 : 200, Cell Signaling Technology), proliferating cell nuclear antigen (PCNA, 1 : 200, Cell Signaling Technology), *α*SMA (1 : 200, Abcam PLC), and CD68 (1 : 200, Abcam PLC) was photographed under light microscopy. In addition, two-colour immunohistochemistry for CD31 (Red), *α*SMA (Green) and CD31 (Green), SM22*α* (Red) was performed using frozen tissue sections, followed by their examination under a fluorescence microscope.

More than ten random fields in each section stained with *α*-SMA were photographed using a Leica 2500 microscope at 100x magnification. Then, area and circumference of *α*-SMA positive vascular medial layer were calculated using Image-Pro Plus 6.0. After that, the *α*-SMA thickness, also named average thickness of vascular medial layer, was calculated as the ratio of area to circumference.

### 2.6. In Situ Terminal Deoxynucleotidyl Transferase-Mediated dUTP-Biotin Nick End-Labelling (TUNEL)

TUNEL Assay Detection of renal cells apoptosis was performed according to the protocol described previously [25] with a TUNEL assay kit (In Situ Cell Death Detection Kit, POD, Roche Company, Germany). Labelled cells (Green) in the kidney were evaluated using fluorescence microscopy. The number of TUNEL-positive nuclei in 6 random different fields per rat was counted. All of these assays were performed blindly, and four rats from each group were evaluated.

### 2.7. Statistical Analysis

Values were presented as the means ± SD. Multiple comparisons between the mean values of the different groups were carried out using one-way ANOVA followed by Bonferroni post hoc test (SPSS software version 19.0). Differences were considered statistically significant when *P* < 0.05.

## 3. Results

### 3.1. Distribution of DPP-4 in the Rat Kidney

**Figure 1:**
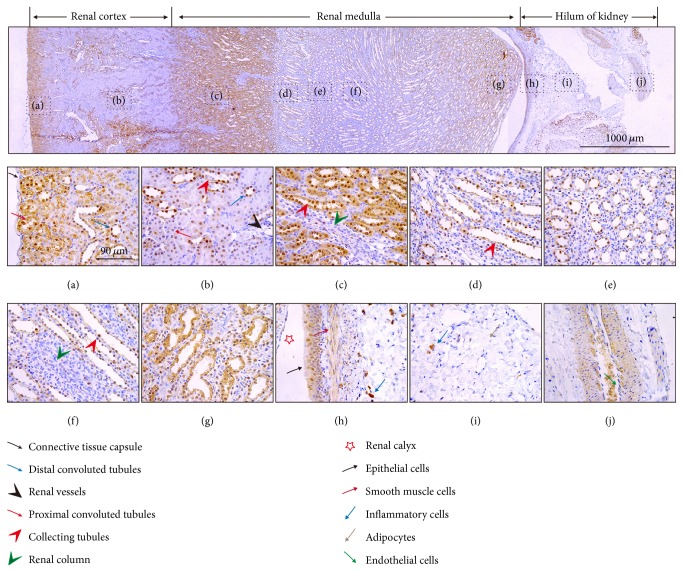
Representative renal immunohistochemical staining for DPP-4.

As shown in Figure 1 on the upper column of panels at low power, the expression of DPP-4 in the kidney was abundant but not homogeneous. According to the results displayed on the lower column of panels at high power, DPP-4 was rarely expressed in the thin connective tissue capsule around the kidney (Figure 1(a)), while it was abundantly expressed in the proximal convoluted tubules and distal convoluted tubules under the renal capsule. The proximal tubules have a taller, pinker epithelium than the thinner epithelium of the distal tubules. However, in the middle of the renal cortex (Figure 1(b)), little expression of DPP-4 was observed in the proximal convoluted tubules, while a rich expression of DPP-4 was still observed in the distal convoluted tubules. From the renal cortex to renal medulla (Figures 1(b)–1(g)), robust DPP-4 expression was observed in the renal collecting tubules, and the protein expression was consistent with the volume of the epithelial cytoplasm. A few DPP-4-positive cells were scattered in the renal column derived from the cortex and extending into the medulla.

In addition, DPP-4 was highly expressed in the epithelial cells of the renal calyx (Figure 1(h)), inflammatory cells (Figures 1(h) and 1(i)), and SMCs (Figures 1(h) and 1(j)), while it was expressed at low levels in vascular ECs (Figure 1(j)) and rarely in adipocytes (Figure 1(i)).

### 3.2. Effect of Sitagliptin and Liraglutide on the Renal Glomerulus Structure and Protein Expression of DPP-4 and GLP-1

**Figure 2:**
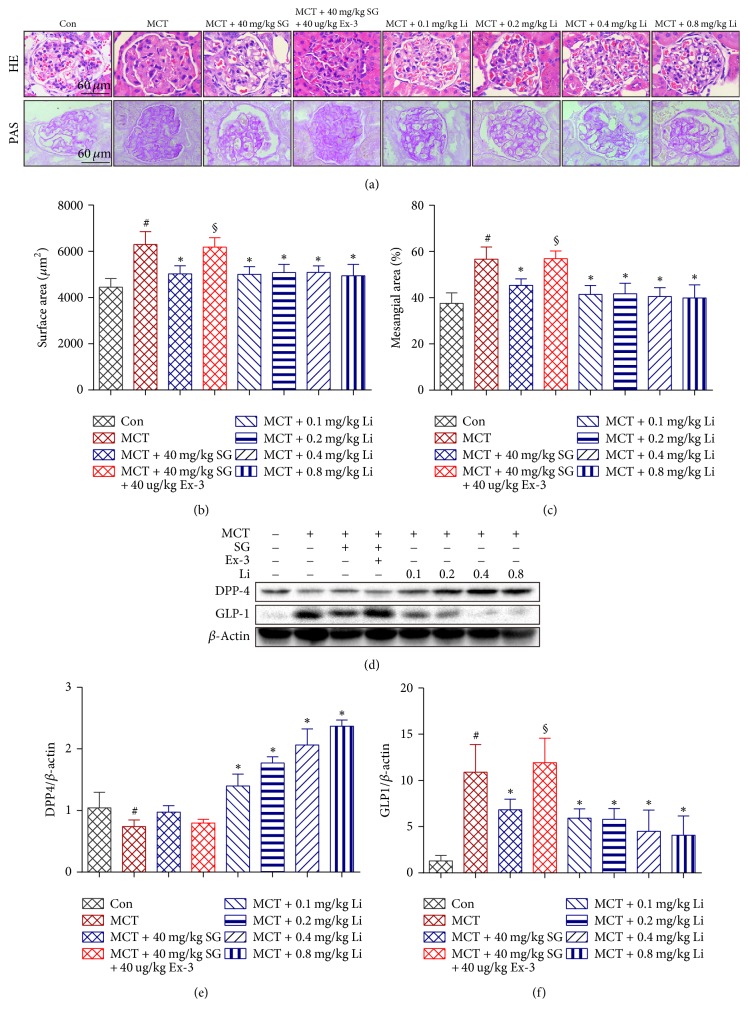
*Effects of sitagliptin (SG), exendin-3 (Ex-3), and liraglutide (Li) on the renal glomerulus structure and protein expression of dipeptidyl peptidase-4 (DPP-4) and glucagon-like peptide-1 (GLP-1) in the kidney during monocrotaline- (MCT-) induced renal injury*. (a) Representative renal histological staining with haematoxylin and eosin (HE) and periodic acid-Schiff (PAS). (b) Graphic analysis of the average glomerulus surface area according to the HE staining. (c) Graphic analysis of the degree of glomerular mesangial expansion according to the PAS staining. (d) Representative western blots of DPP-4 and GLP-1. (e, f) Immunoblot analysis of DPP-4 and GLP-1. Data are expressed as the means ± SD; *n* = 6–8 rats in each group; ^#^*P* < 0.05 versus control (Con); ^*∗*^*P* < 0.05 versus MCT; ^§^*P* < 0.05 versus MCT + 40 mg/kg SG.

Histological sections of SG-treated (DPP-4 inhibitor) kidneys stained with HE and PAS (Figure 2(a)) showed significantly lower glomerular tuft hypertrophy (Figure 2(b)) and mesangial expansion (Figure 2(c)) than kidneys treated with MCT alone, which was blocked by treatment with Ex-3 (GLP-1R antagonist). Li (GLP-1R agonist) effectively and dose-independently attenuated the MCT-induced destruction of the glomerulus structure.

We also examined the expression of DPP-4 and GLP-1 (GLP-1 7-36) in rat renal tissues (Figure 2(d)). Interestingly, expression of DPP-4 was obviously downregulated in the rat kidney treated with MCT compared with its expression in the Con, while treatment with SG partly reversed this change in expression, although the effect was not significant (Figure 2(e)). Additionally, the effects of SG on DPP-4 expression were blocked by Ex-3 to some degree, but no statistically significant trends were observed. However, the expression of DPP-4 was remarkably and dose-dependently upregulated by Li compared with that in the MCT group and was even much higher than in the Con. Meanwhile, the expression of GLP-1 showed the opposite pattern of expression (Figure 2(f)): higher in rats injected with MCT than the Con and lower in rats treated with SG, with the effects of SG treatment blocked by Ex-3 and a dose-dependent decrease in GLP-1 expression in rats injected with Li.

### 3.3. Effect of Sitagliptin and Liraglutide on Renal Injury

**Figure 3:**
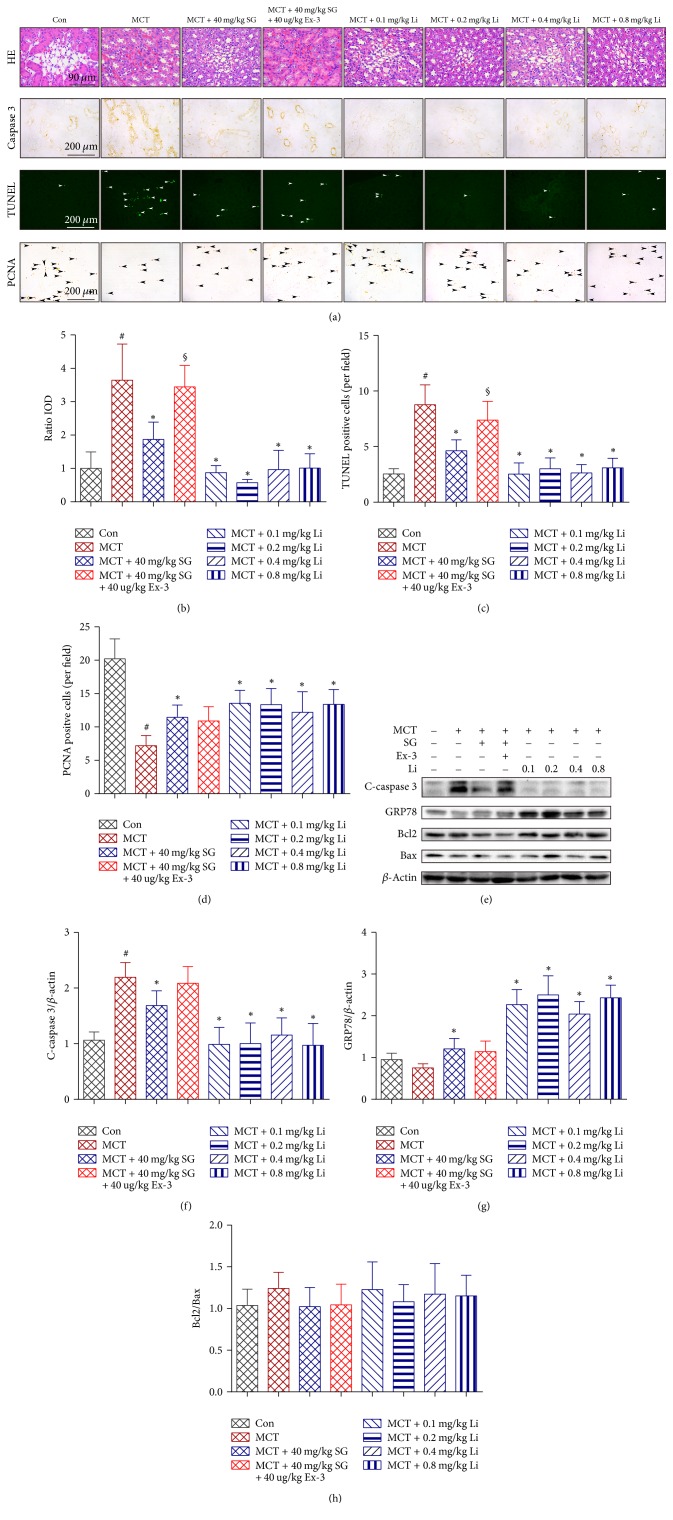
*Effects of sitagliptin, exendin-3, and liraglutide on MCT-induced renal cells apoptosis*. (a) Representative renal histological staining with HE, immunohistochemical staining for caspase 3 and proliferating cell nuclear antigen (PCNA), and TUNEL staining. (b) Graphic analysis of immunohistochemistry for caspase 3. (c) Graphic analysis of TUNEL (×200). (d) Graphic analysis of immunohistochemistry for PCNA (×200). (e, f, g, h) Representative western blots and immunoblot analysis of cleaved-caspase 3 (c-caspase 3), glucose-regulated protein 78 (GRP78), Bcl2, and Bax. Data are expressed as the means ± SD; *n* = 6–8 rats in each group; ^#^*P* < 0.05 versus control (Con); ^*∗*^*P* < 0.05 versus MCT; ^§^*P* < 0.05 versus MCT + 40 mg/kg SG.

In sample HE-stained sections (Figure 3(a)), we also observed vascular thrombosis and expansion of vascular cells in capillary vessels, which is indicative of vascular EC injury and, indirectly, of the remodelling of vessels, in rats treated with MCT or Ex-3 but only rarely in those treated with SG or Li. Furthermore, immunohistochemistry with a primary antibody against caspase 3 and TUNEL staining was performed and displayed in Figure 3(a); here, SG and Li both decreased the MCT-induced apoptosis of renal cells, whereas Ex-3 blocked the effect of SG (Figures 3(b) and 3(c)). Furthermore, SG and Li recovered the normal proliferation of renal cells, while MCT decreased renal cell renewal (Figure 3(d)). However, the above effect of SG was not blocked by treatment with the GLP-1R antagonist Ex-3. The abundance of cleaved-caspase 3 (c-caspase 3) in the renal tissue lysates (Figures 3(e) and 3(f)) was consistent with the above immunohistochemistry results.

In the intracellular signal transduction pathways of GLP-1R, protein survival factors Bcl-2 [26] and GRP78 [27] have been reported to play an important role in cell survival. As shown in Figure 3(e), GRP78 protein expression was lower in rats injected with MCT than the Con but was recovered by SG (Figure 3(g)). Ex-3 partly abolished the effect of SG on the regulation of GRP78. Li not only recovered but also increased the expression of GRP78 compared with that in MCT-treated rats in a dose-independent manner. There was no significant difference in the Bcl2/Bax ratio among the groups in the kidney (Figure 3(h)).

### 3.4. Effect of Sitagliptin and Liraglutide on Renal Fibrosis and the Phenotypic Conversion of Vascular SMCs

**Figure 4:**
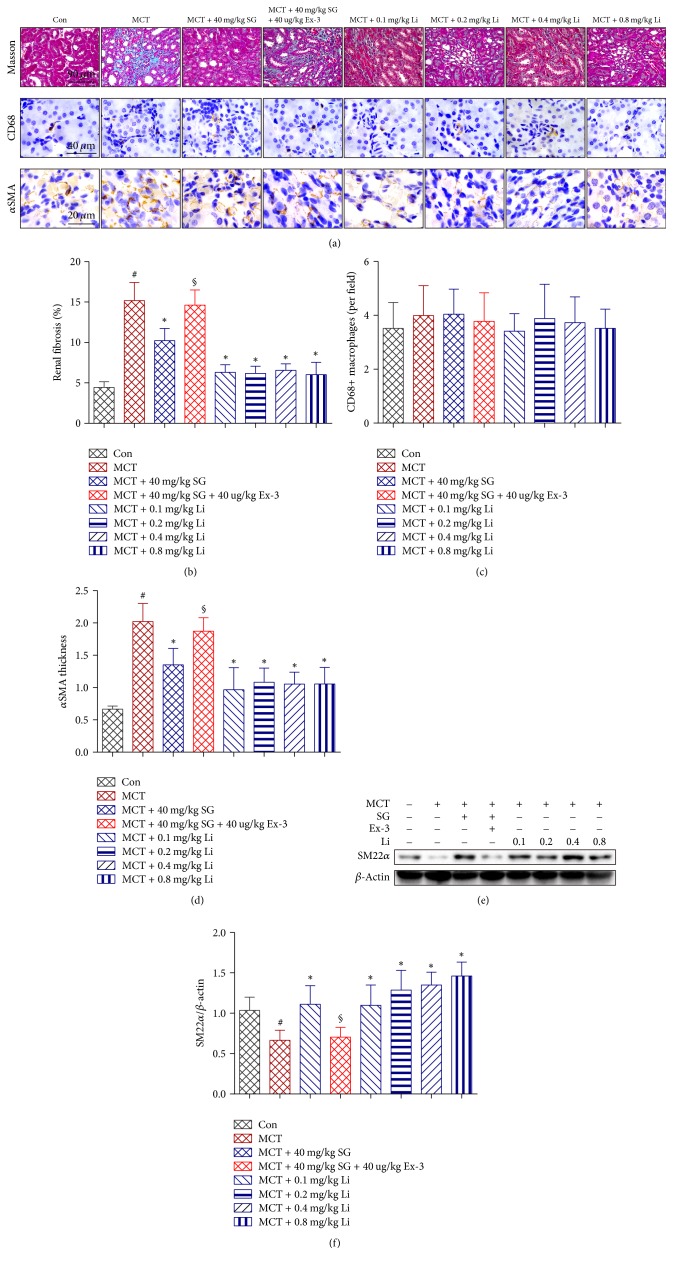
*Effects of sitagliptin, exendin-3, and liraglutide on MCT-induced renal fibrosis and the phenotypic conversion of smooth muscle cells*. (a) Histological staining with the Masson trichrome stain (MTS) and immunohistochemical staining for CD68 and *α*-smooth muscle actin (*α*SMA). (b) Graphic analysis of MTS. (c) Graphic analysis of immunohistochemistry for CD68 (×400). (d) Graphic analysis of *α*SMA thickness. (e, f) Representative western blots and immunoblot analysis of smooth muscle 22 alpha (SM22*α*). Data are expressed as the means ± SD; *n* = 6–8 rats in each group; ^#^*P* < 0.05 versus control (Con); ^*∗*^*P* < 0.05 versus MCT; ^§^*P* < 0.05 versus MCT + 40 mg/kg SG.

To evaluate the effect of SG and Li on renal fibrosis, Masson trichrome staining was used, and the representative results are shown in Figure 4(a). SG and Li markedly inhibited MCT-induced renal fibrosis. In addition, blocking GLP-1R signalling with Ex-3 reduced the protective role of SG in renal fibrosis (Figure 4(b)). We also examined the infiltration of CD68-positive macrophages in renal tissues and found no extensive infiltration in any of the groups (Figures 4(a) and 4(c)).

Since the phenotypic conversion of SMCs could contribute to fibrosis, we performed immunohistochemical analyses of *α*SMA (Figure 4(a)) in renal tissues. SG significantly decreased the thickness of *α*SMA in the microvascular area compared with that in the MCT-treated group, while its effect was abolished by treatment with the GLP-1R antagonist Ex-3. Furthermore, Li dose-independently reduced the hypertrophy of the *α*SMA-positive vascular layer (Figure 4(d)). Expression of SM22*α*, which is expressed at high levels in the contractile phenotype of SMCs but at low levels in the synthetic phenotype, was lower in MCT-treated rats than the Con, and this decrease was reversed by SG. The different doses of Li, except for 0.1 mg/kg/d, had the same effect as SG (Figures 4(e) and 4(f)).

### 3.5. Effect of Sitagliptin and Liraglutide on Endothelial-to-Mesenchymal Transition (EndMT)

**Figure 5:**
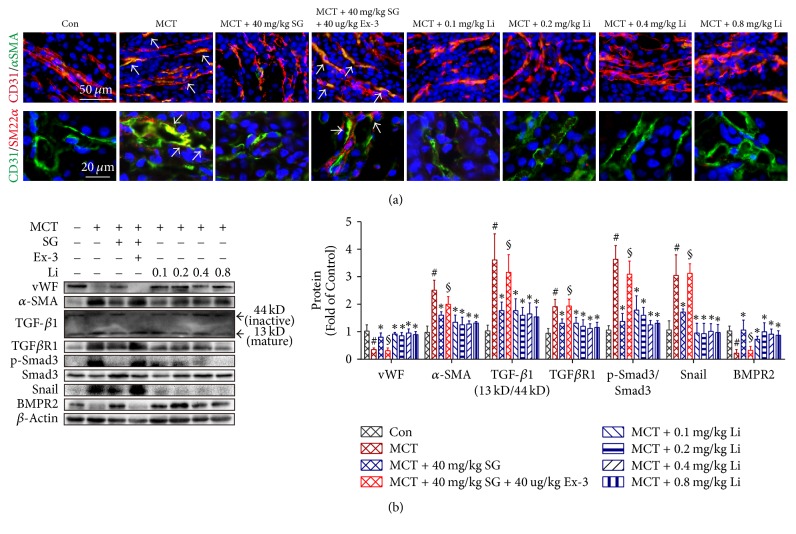
*Effects of sitagliptin, exendin 3, and liraglutide on MCT-induced endothelial-to-mesenchymal transition (EndMT)*. (a) Representative immunohistochemical staining for CD31 (Red), *α*SMA (Green) and CD31 (Green), SM22*α* (Red). (b) Representative western blots and immunoblot analysis of von Willebrand factor (vWF), *α*SMA, transforming growth factor-*β*1 (TGF-*β*1), TGF*β* receptor 1 (TGF*β*R1), phosphorylated Smad3/Smad 3, bone morphogenetic protein receptor type 2 (BMPR2), and Snail. Data are expressed as the means ± SD; *n* = 6–8 rats in each group; ^#^*P* < 0.05 versus control (Con); ^*∗*^*P* < 0.05 versus MCT; ^§^*P* < 0.05 versus MCT + 40 mg/kg SG.

Apart from the phenotypic conversion of vascular SMCs from the contractile phenotype to the synthetic phenotype, EndMT is another essential origin of matrix-producing myofibroblasts. Using immunofluorescence staining against mesenchymal marker *α*-SMA/SM22*α* and endothelial marker CD31, cells undergoing EndMT (double-labelled, yellow, white arrow) were scattered in the kidney of MCT-treated rats and MCT + SG + Ex-3-treated rats, while few double-labelled cells were observed in neither control rats nor rats treated with SG or Li (Figure 5(a)).

**Figure 6:**
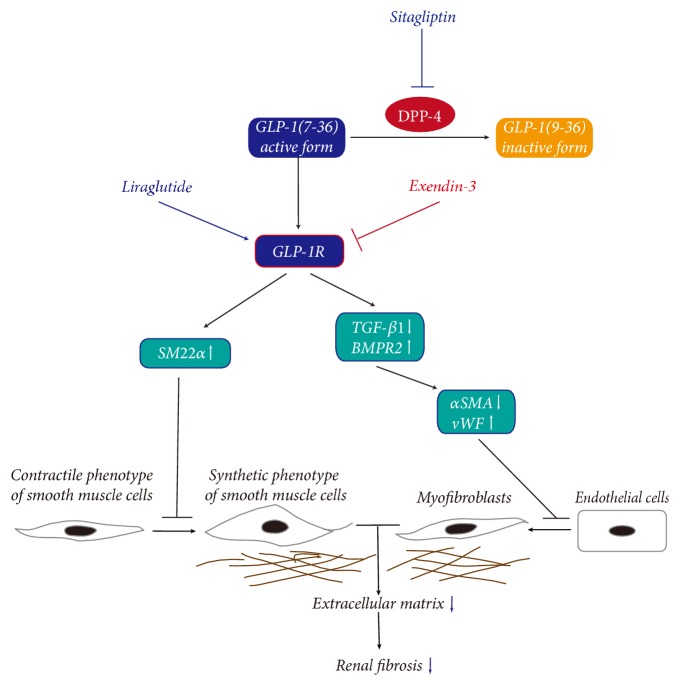
Dipeptidyl peptidase-4 (DPP-4) inhibition with sitagliptin decreases the degranulation of glucagon-like peptide-1 (GLP-1) 7-36 (active form) and promotes the transduction of GLP-1R signalling, which can be blocked by the GLP-1R antagonist exendin-3. The GLP-1R agonist liraglutide can activate GLP-1R signalling directly. In smooth muscle cells (SMCs), the activation of GLP-1R upregulates the expression of smooth muscle 22 alpha (SM22*α*), which is expressed at high levels in the contractile phenotype of SMCs, and inhibits the transition of SMCs from the contractile phenotype to the synthetic phenotype. The extracellular matrix derived from the synthetic phenotype of SMCs is then obviously reduced. In endothelial cells, activated GLP-1R signalling can upregulate bone morphogenetic protein receptor type 2 (BMPR2) expression and reduce transforming growth factor-*β*1 (TGF-*β*1)/Smad3 signalling, followed by inhibiting Snail expression. Then, the protein expression of endothelial marker von Willebrand factor (vWF) increases, while that of the mesenchymal marker *α*-smooth muscle actin (*α*SMA) decreases. After that, the endothelial-mesenchymal transition (EndMT) programme is blocked, the extracellular matrix derived from myofibroblasts is reduced, and, ultimately, renal fibrosis is attenuated.

For quantitative analysis of EndMT in kidney, the contents of the total protein from renal tissue lysates were examined by western blot analysis (Figure 5(b)). Consistent with the results above, expression of the endothelial marker vWF was downregulated, while that of the mesenchymal marker *α*SMA was upregulated in rats challenged with MCT compared with those in the Con. These effects were reversed by both SG and Li treatment. Combined treatment with Ex-3 abolished the action of SG not only on vWF expression but also on *α*SMA. In addition, we also evaluated the expression of TGF-*β*1/TGF*β*R1/Smad3 and Snail, which are involved in the EndMT programme, as well as BMPR2, which can protect cells from EndMT. As expected, mature TGF-*β*1 (13 kD)/inactive TGF-*β*1 (44 kD), TGF*β*R1, p-Smad3/Smad 3, and Snail increased in MCT-treated rats and Ex-3-treated rats and decreased in SG- and Li-treated rats. In contrast, BMPR2 showed the opposite changes in expression, including its upregulation in rats treated with SG, which was abolished by Ex-3. These results above were briefly presented in Figure 6.

## 4. Discussion

Both alleviating kidney fibrosis and restoring normal kidney structure are fundamental processes to explore for developing therapies to prevent patients from progressive kidney disease. Myofibroblasts, originating from diverse sources, play a key role in renal structural alterations including renal fibrosis [28–30]. Although EMT has been demonstrated to be one of the most important sources of myofibroblasts and to be responsible for kidney interstitial fibrosis in numerous studies [31–33], EndMT has also been identified as another source of myofibroblasts [28, 29, 34] and is more important than EMT [6]. EndMT, combined with other forms of endothelial dysfunction including apoptosis, alterations in the endothelial barrier permeability and impairment of endothelium-dependent vasorelaxation [35], participates in renal microcirculation lesions. Apart from EndMT, the transition of SMCs from the contractile to the synthetic phenotype is another structural vascular alteration that can result in microcirculation lesions and the resulting renal fibrosis by producing fibrillar collagens, contractile proteins, and excessive matrix deposition. Renal fibrosis resulting from microcirculation lesions, including the phenotypic conversion of renal ECs and SMCs, results in the loss of renal function and, eventually, renal failure. Taken together, renal fibrosis resulting from microcirculation lesions, including the phenotypic conversion of renal microvascular cells, was the focus of the present study and requires more attention in the future.

We took advantage of MCT to induce renal microcirculation lesions described by Kurozumi et al. [36] and several other researchers [37, 38]. In the present study, just as in lung tissues [39], intraperitoneal injection of MCT successfully resulted in renal cell apoptosis and EndMT in renal tissues without extensive infiltration of inflammatory cells, such as CD68+ macrophages, or excessive proliferation and infiltration of fibroblasts. Compared with the infiltration of numerous inflammatory cells and fibroblasts in renal lesions induced by lipopolysaccharide (LPS) [40], cisplatin [41], high-fat diet [42], angiotensin II [43], diabetes [44], and so forth, renal lesions induced by MCT are characterized by renal microcirculation lesions such as glomerular tuft hypertrophy, mesangial expansion, phenotypic conversion of SMCs, and EndMT without an extensive inflammatory response.

Consistent with the antiapoptotic action of DPP-4 inhibition in renal injury induced by tacrolimus [16] and ischaemia-reperfusion [19], sitagliptin effectively decreased renal cell apoptosis in the present study. This antiapoptotic effect was mediated by GLP-1/GLP-1R signalling since it was blocked by the GLP-1R antagonist exendin-3. Furthermore, activating GLP-1R with Li resulted in the same effect on apoptosis. According to previously published results, protein survival factors Bcl2 [26] and GRP78 [27] are involved in the intracellular signal transduction pathways of GLP-1R [45]; therefore, we examined the expression of Bcl2 and GRP78 to further investigate the underlying mechanism. On the one hand, there was no significant difference in the Bcl2/Bax ratio among the groups. On the other hand, GRP78 expression was downregulated in MCT-treated rats but was recovered by treatment with sitagliptin and dose-independently increased by liraglutide. The upregulation of GRP78 expression with a GLP-1 analogue has been shown to improve rat cortical neuron survival [27] and enhance the unfolded protein response (UPR) and reduce hepatocyte steatosis, thereby improving survival [46]. Taken together, GLP-1 mediated the antiapoptotic effect of DPP-4 inhibition on MCT-induced renal injury through upregulating GRP78 expression; however, the crosstalk between GLP-1/GLP-1R signalling and the UPR requires more exploration.

SMCs were briefly divided into two phenotypes: contractile phenotype (differentiated SMCs) and synthetic phenotype (dedifferentiated SMCs) [47, 48]. In response to a stimuli associated with vascular injury and diseases (such as atherosclerosis, hypertension, aortic aneurysm formation, and postangioplasty restenosis), SMCs will switch from contractile phenotype to synthetic phenotype and may migrate, proliferate, and secrete extracellular matrix (ECM) and remodelling factors, among other behaviours [49, 50]. Synthetic SMCs transformed from the contractile phenotype promote both vascular structural modification [51, 52] and airway remodelling [53, 54] due to their abnormal proliferation, migration, and production of ECM. However, they have rarely been noted in renal disease. In the present study, we analysed the phenotypic conversion of vascular SMCs by detecting the thickness of the *α*SMA-positive SMC layer and protein expression of SM22*α*, which is abundant in the contractile phenotype of SMCs but low in the synthetic phenotype. Here, our results demonstrated that the phenotypic conversion of SMCs contributed to MCT-induced renal microcirculation lesions, which were reduced with the use of sitagliptin or liraglutide. The ability of sitagliptin to regulate the phenotypic conversion of SMCs was dependent on GLP-1/GLP-1R signalling since it was blocked by treatment with the GLP-1R antagonist exendin-3. In brief, DPP-4 inhibition attenuated MCT-induced renal lesions partly by recovering SMCs from the synthetic phenotype to the contractile phenotype in a GLP-1-dependent manner.

Apart from the phenotypic conversion of SMCs, EndMT is another manifestation of renal microcirculation lesions and contributes to renal fibrosis [55]. We found that MCT upregulated the expression of the mesenchymal marker *α*SMA, downregulated the expression of the endothelial marker vWF, and finally increased the number of CD31-*α*SMA double-positive cells in the kidney, indicating that MCT induced EndMT in the kidney. Sitagliptin decreased EndMT through GLP-1/GLP-1R signalling as the effect of sitagliptin was blocked by the GLP-1R antagonist exendin-3. In addition, activating GLP-1/GLP-1R signalling with liraglutide also reversed MCT-induced EndMT. To further explore the underlying molecular mechanism, TGF-*β* family signalling, including BMPR2 signalling and TGF-*β*1/Smad3 signalling, was investigated. Numerous studies have shown that TGF-*β*2R signalling is involved in EndMT in different disorders such as vein remodelling [56], atherosclerosis [57], cardiac fibrosis [30], and pulmonary hypertension [58], as well as renal injury [59, 60]. The EndMT programme induced by TGF-*β*2R signalling can be inhibited by activating BMPR2 signalling [30, 58]. As expected, sitagliptin blocked the MCT-induced maladaptive responses including downregulated BMPR2 and upregulated TGF-*β*1, eventually preventing the cells from undergoing the Snail-mediated EndMT programme. Taken together, DPP-4 inhibition prevented normal ECs from underdoing MCT-induced EndMT in a GLP-1R-dependent manner by regulating TGF-*β* family signalling.

In contrast to the upregulation of DPP-4 expression observed in renal tissues injured by diabetes [60], obesity [61], and free fatty acid-bound albumin [62], the present study showed that DPP-4 expression was downregulated in rat renal tissues injected with MCT. According to the DPP-4 immunochemistry, epithelial cells and inflammatory cells were the most important sources of DPP-4 in kidney. Therefore, increased expression of DPP-4 in other models of renal injury might result from extensive infiltration of DPP-4-positive inflammatory cells. However, in the present study, DPP-4-positive inflammatory cells, such as CD68-positive macrophages, did not infiltrate the renal tissues, which may explain the lack of an increase in DPP-4 expression in MCT-treated rats. The downregulation of DPP-4 in rats challenged with MCT might have resulted from a renal self-protective programme, but this requires further investigation. Meanwhile, the expression pattern of the substrate of DPP-4, GLP-1, in renal tissues was opposite of that for DPP-4 since GLP-1 can be degraded by DPP-4.

In conclusion, DPP-4 inhibition attenuated MCT-induced renal fibrosis by reducing renal cell apoptosis via increasing the protein expression of survival factor GRP78, inhibiting the phenotypic conversion of renal microvascular SMCs, and inhibiting EndMT via upregulating BMPR2 and downregulating TGF-*β*1. Furthermore, the protective actions of DPP-4 inhibition were GLP-1/GLP-1R signalling-dependent. Therefore, a targeted therapy aimed at inhibiting DPP-4 activity or activating GLP-1R signalling may be a prospective approach in the management of renal disorders such as renal microcirculation lesion-induced fibrosis.
